# Expression of miR-302 in human embryo derived from in-vitro matured oocyte

**DOI:** 10.18502/ijrm.v17i6.4812

**Published:** 2019-07-29

**Authors:** Parvin Dorfeshan, Marefat Ghaffari Novin, Mohammad Salehi, Fatane Farifteh

**Affiliations:** ^1^Department of Social Medicine, Faculty of Medicine, Jundishapure University of Medical Sciences, Ahvaz, Iran.; ^2^Department of Biology and Anatomical Sciences, Faculty of Medicine, Shahid Beheshti University of Medical Sciences, Tehran, Iran.; ^3^Infertility and Reproductive Health Research Center, Shahid Beheshti University of Medical Sciences, Tehran, Iran.; ^4^Cellular and Molecular Biology Research Center, Shahid Beheshti University of Medical Sciences, Tehran, Iran.; ^5^Department of Biotechnology, School of Advanced Technologies in Medicine, Shahid Beheshti University of Medical Sciences, Tehran, Iran.

**Keywords:** miR-302, Embryonic development, Ovarian stimulation, In-vitro maturation, Intracytoplasmic sperm injection.

## Abstract

**Background:**

The expression of miR-302 over the period of early embryogenesis could possibly regulate the maternal transcript clearance. Zygotic transcription activation is mostly related to maternal messages degradation.

**Objective:**

In this study, the effects of in-vitro maturation technique (IVM) on the expression of miR-302 in human embryo produced from immature and mature human oocytes (matured in vitro and in vivo, before sperm exposure) obtained from females under gonadotrophin therapy were evaluated for assisted reproduction.

**Materials and Methods:**

Immature oocytes were cultured in vitro. The injection of oocytes-producing polar bodies was given using fresh sperm. Then, the embryo quality score was assessed in the IVM group compared with the control group. In both the groups, embryos with normal morphology were included in the molecular study. Only one blastomere was removed from three-day embryos and then the embryos were frozen. The expression of miR-302 in embryos was measured through quantitative real-time polymerase chain reaction.

**Results:**

Our data showed a significant reduction of miR-302 expression in the IVM group vs. the control group (p = 0.02). The embryo quality score showed a significant difference between the two groups (p = 0.01).

**Conclusion:**

The present study demonstrated that the IVM process had a negative effect on the expression level of miR-302 in human pre-implantation embryos. Considering the major role of expression miR-302, a reduced potential in miR-302 expression could be related to a decrease in the early embryonic development.

## 1. Introduction

The complete maturation of oocytes is very substantial in supply and accumulation of maternal transcription factors (TFs) particularly at the final step of the oocyte growth phase (the process in which germinal vesicle (GV) progresses to the metaphase II stage) (1). The recovery of immature oocytes, and subsequent in-vitro maturation (IVM), of such oocytes could be established as a procedure to cure women with infertility or to obtain more oocytes or more chances for infertile couples. No aspect of this therapy technique was known so far (2). There are many uncertainties over IVM culture, complete and comprehensive of oocyte maturation; such as nuclear, membrane, and cytoplasmic maturation. For the time of early stages of embryogenesis, development is primarily controlled by maternal parameters contributed by the egg cytoplasm, but the zygotic nuclear genome is inactive. Consequently, the activation of this genome occurs, products of the embryonic gene are moved, and the clearance of maternal parameters occurs. This developmental control transfer is known as maternal-to-zygotic transition (MZT) and is coupled with arising embryonic genome activation (3).

During MZT and over cellular reprogramming, the history of the cell is deleted to make possible the formation of new cellular states via particular transcription parameters (zygotic state or pluripotency). Despite the variety of mechanisms recognized among diverse species to regulate the expression of a gene, uniting themes, such as the roles of central TFs and microRNAs (miRNAs) to direct the MZT emerge, miRNAs were indicated to control temporal gene expression by downregulating mRNAs transcribed over earlier developmental steps (4-7). However, it is important to note that such miRNAs might play significant roles in the maternal transcript clearance in mammals. There are numerous lines of evidence suggesting that such miRNAs not only contribute to pluripotency but also have key roles in cellular reprogramming. Importantly, miR-430 (one of the earliest and most highly transcribed genes originated from the zygotic genome (8-11)) and miR-427 have abundant expression during MZT in zebrafish (12, 13) and Xenopus (14, 15), respectively. miR-302 and miR-372 are its orthologous in humans (16-19).

According to the previous studies, maternally prepared parameters have the ability to activate zygotic gene transcription through the function of TFs involving the early embryonic genome. In addition, zygotic genes, such as miRNAs, play an important role in maternal RNA clearance. The failure in the activation of the zygotic genome through the inhibition of maternal TF activity leads to stabilized maternal program and arrested development. miR-302 and miR-372 expression occurs over early development stages in humans and primates (20).

Based on the aforementioned information, the present study was designed to assess the effects of oocyte culture on miR-302 expression in the human embryo. The aim of this study was to present the first expression library of miRNAs within the human embryos derived from the IVM technique to provide a necessary starting point for future investigations to improve the IVM.

## 2. Materials and Methods

### Patients

In the present research, two different experiments were studied. The control group included 16 couples who underwent intracytoplasmic sperm injection (ICSI) cycles and then pre-implantation genetic diagnosis (PGD) was done for them. In the control group, after embryo transfer, the excess of normal biopsied embryos that were donated for scientific research. The IVM group included 36 couples who underwent Repeated Implantation Failure (RIF) treatment and immature oocytes used at the GV stage in the present study. The exclusion criteria were any infertility with female factor as well as male factor. The age of the female and male patients were less than 35 and 45 yr old, respectively.

### Controlled ovarian stimulation

For controlled ovarian stimulation, all the patients received the standard long protocol of pituitary suppression with gonadotrophin-releasing hormone agonist. Hypophyseal suppression of patients was started by administering 0.1 mg of Diphereline (triptorelin; Ipsen Pharma, Paris, France) or Decapeptyl (triptorelin; Ferring Pharmaceutical, Germany) in the mid luteal phase of the previous cycle. Subsequently, we decreased gonadotropin-releasing hormone analog dose to 0.05 mg until the administration day of human chorionic gonadotropin (HCG). The patient's ovaries were stimulated with recombinant follicle-stimulating hormone (FSH) (Gonal-F; Merck Serono, Germany). The initial doses were determined and administrated based on the patient's age as well as basal serum FSH and estradiol levels. The patients received transvaginal ultrasound. HCG (10000 IU, IBSA, Lugano) was administrated. After 36-38 hr of the HCG injection, follicles larger than 11 mm were punctured, using transvaginal ultrasonography.

### Oocyte insemination and evaluation of embryo quality score

GV oocytes were collected. The oocytes were denuded with 1% hyaluronidase enzyme (Life Global, Guilford, CT, USA), gently aspirated using a hand-drawn glass pipette, and incubated under mineral oil (Irvine Scientific, USA) for a maximum of 1-hr in 6% CO${}_{2}$ at 37°C (Memmert, Germany). GV oocytes were observed using an inverted microscope (SM2800, Nikon) at ×200 magnification. Our criteria for choosing GV oocytes were based on the presence of a prominent nucleus in the homogeneous cytoplasm with any defect in the overall appearance of oocytes. The GV oocytes were maintained in a drop containing 20-30 µL of commercial IVM media (Sage Media, USA, Oocyte Maturation Medium) supplemented with 75 IU/ml FSH and 75 IU/ml Luteinizing hormone for 24-30 hr based on the manufacturer's protocols.

Oocytes showing liberated polar bodies were injected by sperm, based on the World Health Organization criteria (21, 22). The injected oocytes were evaluated following 16-18 hr for the appearance of pronuclei according to our routine screening procedure. Zygotes were maintained in the culture media for further 24-30 hr. Three days after ICSI, the embryos were evaluated and scored for their cleavage and quality. The morphological features of embryos were determined according to a previously published study (23).

### Blastomere biopsy

Three days after ICSI, the embryos moved into microdrops of biopsy media (LG PGD BIOPSY medium, Life Global) that were under mineral oil. Zona pellucida drilling was performed mechanically and one blastomere was biopsied for PGD analysis. The embryo was transferred into a drop for embryo vitrification. The embryo was maintained into an equilibration solution (Kitazato BioPharma Co., Ltd., Japan) for 15 min at room temperature (20-27°C), after an initial shrinkage and recovery. Subsequently, the embryo was aspirated and placed in the vitrification solution (Kitazato BioPharma Co., Ltd., Japan) at room temperature for a period of 1 min and then placed on a cryotop with a minimum volume of VS solution (24). Cryotop quickly plunged them into liquid nitrogen.

### RNA isolation and cDNA synthesis

The samples were applied to isolate RNA and synthesize cDNA, followed by quantitative real-time (qRT)-polymerase chain reaction (PCR) analysis. Extraction of RNA, synthesis complementary of DNA (cDNA) and analysis were done using the protocol explained in the previous studies. (25-27). In brief, the samples were pipetted into Eppendorf tubes consisting of 1.5 µL lysis buffers. cDNA was synthesized by the addition of 2 µL poly N, 2 µL mir-302 primer, and 5 µL nuclease-free water to each of 2 µL embryo samples. The samples were then placed in a Bio-Rad thermocycler for 5 min at 75°C until the reaction was complete. Subsequently, the tubes were placed on ice, and 5× RT buffer (5 µL), 200 u RT enzyme (1 µL), 10mM dNTP (3 µL), and 10 u RNase inhibitor (0.25 µL) were added to the reaction for the reverse transcription (RT). RT reaction was performed at 25°C for 10 min, 37°C for 15 min, 42°C for 45 min, and 72°C for 10 min. Afterward, the samples were kept at 4°C overnight.

Table I indicates the primer sequences used in this study for qRT-PCR to study the frequency of the level of mir-302 via Rotor-Gene Q instrument (Qiagen, Turnberry Lane, Valencia, CA, USA). We performed RT-PCR reactions and reaction conditions (denaturation, amplification and extension) based on the protocol described in the previous study (27). A single gene-specific peak of different amplification reactions was verified by melting curve analysis. One reference gene, Snord, was used for the normalization of the RT-PCR as an endogenous internal gene. Additionally, we performed three replications and normalized fold-change in the mRNA level for each sample to that of endogenous internal mRNA levels (25, 27).

**Table 1:** Primers used in this study for quantitative real-time PCR


**Gene name**	**Primer sequence (50-30 orientation)**
miR-302a	Forward: GTC GTA TGC AGA GCA GGG TCC GAG GTA TTC GCA CTG CAT ACG ACT CAC Reverse: CCG CTA AGT GCT TCC ATG TTT TG
SNORD	Forward: ATC ACT GTA AAA CCG TTC CA Reverse: GTC GTA TGC AGA GCA GGG TCC GAG GTA TTC GCA CTG CAT ACG ACA ACC TC

### Ethical consideration

This study was approved by the Ethics Committee of the Shahid Beheshti University of Medical Sciences, Tehran, Iran (SBMU.REC.1393.78). In current research, written and verbal informed consent was achieved from the couples who had undergone ICSI/PGD according to standard protocols.

### Statistical analysis

All statistical analyses were carried out using the Statistical Package for the Social Sciences software, version 22 (SPSS, Chicago, IL, USA). The embryo quality score was compared using the non-parametric analysis test (Mann–Whitney U test). The results are expressed as means % ± SEM. The relative gene expression level of the related gene among embryos from two groups was analyzed by REST 2009 software (Qiagen). p < 0.05 were considered to be statistically significant.

## 3. Results

Germinal vesicle oocytes were collected from 36 couples undergoing treatment. The frequency of harvested GV oocytes ranged from 3 to 6. We used the results of 16 couples undergoing ICSI-PGD cycles (Table II). After the development of the embryos at the 8-cell stage, the embryos having normal morphological features were chosen for molecular studies. Embryo quality score reduced significantly in the IVM group when compared with the control (p = 0.01) (Table II).

**Figure 1:**
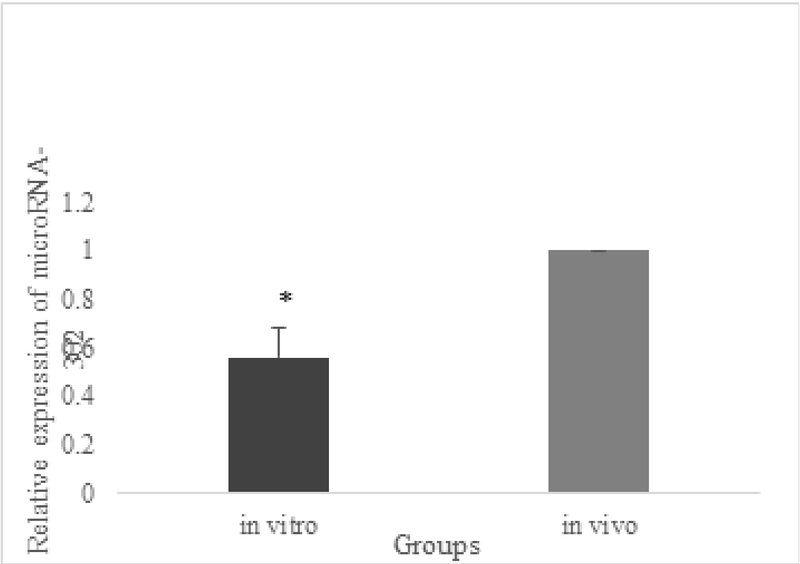
miRNA transcript relative quantification. Relative expression of mRNA of miR-302a in 8-cell stage of human embryos in the IVM group and control group demonstrated a statistically significant difference between the two groups. The mRNA level of each gene was analyzed using quantitative real time-PCR. In addition, the mRNA levels of each sample were normalized to the level of Snord mRNA. The data are presented as mean ± SEM. (*) p < 0.05. The relative gene expression level of the related gene among embryos from two groups was analyzed by REST 2009 software (Qiagen).

Real-time PCR was performed to assess the quantitative expression of the studied gene. The miR-302 levels were measured by qRT-PCR in 8-cell stage embryos derived from ICSI using freshly prepared sperm. According to the criteria explained by the World Health Organization, sperm parameters (such as concentration, motility and morphology) were determined (21). The morphological features of sperms were also determined using the Kruger strict criteria (22). The comparison of the relative transcript abundance miR-302 mRNA in embryos derived from ICSI indicated a significant difference at the levels of this micoRNA, during human early developmental stages, between the IVM group and the control group (p = 0.02). Figure 1 indicates the relative levels.

**Table 2:** Demographic characteristics of all evaluated ICSI cycles


**Characteristic**	**Group**	
	**IVM oocytes**	**Control oocytes**
Number of cycles	36	16
Female age (yr) (Mean ± SEM)	30.1 ± 1.2	28.9 ± 1.6
No. of injected oocytes	124	121
Embryo quality score (Mean ± SEM)	1.86 ± 0.67	2.30 ± 0.31
Note: ICSI: Intracytoplasmic Sperm Injection IVM: In-vitro Maturation		

## 4. Discussion

The purpose of the present study was to investigate the effects of the IVM technique on the expression of miR-302 in human embryos grades A and B, produced from immature and mature oocytes (matured in vivo and in vitro, before sperm exposure) and obtained from females under gonadotrophin therapy for assisted reproduction. The main aspect of the present study was to assess one of the major miRNAs over early human embryonic development in the Assisted Reproductive Technique (ART). Our findings demonstrated that miR-302 expression diminished significantly in the IVM group when compared with the control group (p = 0.02).

Previously published studies in this field demonstrated the high expression of miR-302 in the development of an early human embryo. The results obtained from those studies, indicating increased expression over the early period of the embryo, document a putative role for such miRNAs already identified in embryonic stem cells (ESC), demonstrating their major roles in maintaining pluripotency (20). miRNAs, small non-coding RNA molecules, regulate genes in numerous biological systems, including the oocyte and embryo (28). Previous studies indicated that the stem-cell factors, such as Oct4, link miR-302 as a promoter and stimulate its expression in ESCs (6). Epigenetic reprogramming events have the ability to stimulate the expression of ESC specific genes, particularly Oct4, Sox2, and Nanog, in turn further induce the expression of miR-302 for the production of a positive feedback loop cycle having a key role in maintaining somatic cell reprogramming (SCR) (29). It has been reported that several possible miR-302 targets regulate the cell cycle. Therefore, the regulation of cell-cycle development mediated by miR-302 may be ascribable to directing multiple mRNAs. This suggested that miR-302a might be coordinately directing regulators of multiple G1 phases toward cyclin D1 and Cdk4 for serving as a negative regulator of G1 (6). Particularly, this emphasize the main role of miR-302a in the proliferation of EC cells. Interestingly, miR-294 in mice and miR-302/372 in humans are plentifully stated in ESCs and embryonic tissues (20, 30, 31). Collectively, miR-302 functions in a single-cell range, such as contributing to erase the history of cells to make easy the formation of new cellular states by specific TFs (zygotic state) and pluripotency, have important roles in cell reprogramming and cell cycle regulators (6, 17, 29).

Reviewing how mammalian oocytes evolve, we can find that they obtain a sequence of abilities over follicular progress (growth of oocyte and maturation of cytoplasm) having important effects on fertilization and following early embryonic progress (2, 28). The final preparation of oocytes for fertilization is carried out during oocyte maturation (from the GV to metaphase II stage) (2). These factors are applied to maintain the early phase of embryonic progresses before the beginning of embryo DNA transcription (32, 33). During the cytoplasmic maturation synthesis, essential maternal materials including maternal TFs are synthesized that involve the early embryonic genome and contain miR-302 in human early embryonic development. miR-302 is one of the human embryonic genes whose expression is govern by a wide variety of factors, containing various functions in embryonic and stem cells (2). Therefore, based on our findings, a decrease in the expression of miR-302 (considering its main role) in the embryo derived from in-vitro matured oocyte can be related to a decrease in early embryonic development so that this reduction cannot be overlooked.

Generally, the routine assessment of oocyte morphology using the phase-contrast microscopy is a key predictive marker used for oocyte quality, therefore mature oocytes, with full nuclear maturation followed by the first polar body extrusion, are currently utilized to measure the success or failure of a given ART program (34). Initial studies emphasize to “nuclear maturation could occur suddenly subsequent to the culture in vitro of animal and human immature oocytes” (2). Nevertheless, the developmental competence after fertilization in the later stages of these oocytes is doubtful. Culture conditions considerably affect the maturation of in-vitro oocytes. Not only normal morphology and nuclear maturation of oocyte but also many factors are involved and active in the early progress of the embryo development, which is neglected in the IVM method. Therefore, due to the lack of the probable clinical methods, cytoplasmic maturation is ignored in ART programs, and the embryo development sequences in the next phases remain unknown and more studies are required in this area.

## 5. Conclusion

The present study indicated that IVM process had a negative effect at the expression level of miR-302 in human pre-implantation embryos (despite the normal morphology for these embryos). Therefore, these results could change several events involved in establishing pluripotency and in improving the activation of zygotic transcription in the development of the embryo, requiring further and advanced studies at the molecular level and improvement of culture media for in-vitro maturation.

##  Conflict of Interest

Authors declare that they have no conflict of interests.
